# Iliac vein aneurysms: a comprehensive review

**DOI:** 10.15171/jcvtr.2019.01

**Published:** 2019-02-19

**Authors:** Sina Zarrintan, Niki Tadayon, Seyed Moahammad Reza Kalantar-Motamedi

**Affiliations:** ^1^Division of Vascular & Endovascular Surgery, Department of General & Vascular Surgery, Shohada-Tajrish Hospital, Shahid Beheshti University of Medical Sciences, Tehran, Iran; ^2^Phlebology Research Group, Shahid Beheshti University of Medical Sciences, Tehran, Iran

**Keywords:** Iliac Vein, Aneurysm, Arteriovenous Fistula, Venous Malformation

## Abstract

***Introduction:*** Iliac vein aneurysm is a rare clinical entity. Iliac venous tract is the least commonlocation for venous aneurysms. There are a few cases of common, external and internal iliac veinaneurysms in the literature. However, undiagnosed and ruptured iliac venous aneurysms couldhave hazardous consequences. Herein, we reviewed all literature cases of iliac vein aneurysms.Their potential diagnostic and therapeutic challenges are discussed.

***Literature Review:*** Following a systematic search, 50 cases of iliac venous aneurysms wereidentified. We used MEDLINE [1900-March 2018] and EMBASE [until March 2018]. MeSHterms of iliac vein/veins/venous, hypogastric, inferior vena cava and aneurysm/aneurysms wereused. Fifty patients with venous aneurysms located in common, external or internal iliac veinswere found in our systematic search. Seventeen patients were female (35.4%) and 31 patients weremale (64.6%). The age range was 13 to 70 years of age. The aneurysm was located in right side in17 patients (34%). It was located in left side in 29 patients (58%) and it was bilateral in 4 patients(8%). The aneurysm was located in common, external and internal iliac veins in 15 (30%), 31(62%) and 4 (8%) patients respectively. The aneurysm was due to a previous arteriovenousfistula (AVF) in 19 patients (38%) and of them, 16 patients (32%) had a history of AVF resultingfrom a previous trauma. 29 patients (59.2%) underwent open surgical treatment. Five patients(10.2%) underwent endovascular treatment. One patient (2.0%) underwent hybrid treatment.Conservative treatment was used in 14 patients (28.6%).

***Conclusion:*** Iliac vein aneurysms are extremely rare. Its diagnosis necessitates precise clinicalsuspicion and the treatment is based on patients’ clinical scenario and radiological features. Bothopen and endovascular techniques could be feasible. Iliac vein aneurysms are more commonin men. Left sided aneurysms are more common. The most common anatomic location isexternal iliac vein. The most common cause of iliac aneurysms is dilatation of vein secondary toa traumatic AVF.

## Introduction

Iliac vein aneurysm is an extremely rare entity. Iliac system is the least frequent location for venous aneurysms.^1^ Thus, review on literature cases and discussion on its etiology, presentation, diagnosis and management is of potential clinical interest. Iliac aneurysms could be primary or secondary to an underlying cause. Both types are rare. The most common secondary cause of an iliac aneurysm is arteriovenous fistulas (AVFs).^2,3^

Presentation of iliac venous aneurysms is diverse and unclear due to its rarity.^4^ Duplex ultrasound, CT venography, magnetic resonance venography and conventional venography are diagnostic tests to identify iliac vein aneurysms and to plan specific management. However, simple test has not yet been gold standard.^5^ Patients with venous aneurysms are usually asymptomatic but complications can occur. These include thrombosis, rupture, embolization and mass effects.^6^ Thus, diagnosis and treatment of this rare entity is of potential clinical importance.

The largest review on iliac vein aneurysms in the literature was done by Ysa et al in 2008.^1^ They reviewed 23 cases of iliac vein aneurysms. Despite their extensive analysis, low number of cases made it difficult to reach to conclusion on its pathogenesis, diagnosis and management. Herein, we reviewed all the cases of iliac vein aneurysms until March 2018. Comprehensive descriptive analysis is presented. Presentation, etiology and management of this rare entity is discussed. Anatomical locations, open surgical and endovascular management of iliac vein aneurysms are presented. This is the first review to collect all cases of iliac vein aneurysms.

## Literature Review

### Search strategy

A systematic search was conducted to perform this comprehensive review. We used MEDLINE [1900-March 2018] and EMBASE [until March 2018]. MeSH terms of iliac vein/veins/venous, hypogastric, inferior vena cava and aneurysm/aneurysms were used. Fifty patients with venous aneurysms located in common, external or internal iliac veins were found in our systematic search. An unrestricted search strategy was used. Full-texts of the reported cases were extracted using institutional access to publishers’ websites.

### Statistical analysis

The reported cases were presented by frequency and percent. The age of the reported cases was presented by mean ± SD. Independent sample t-test was used to compare continuous data. Chi-square test was used to compare categorical data. All analyses were conducted by the Statistical Package for Social Sciences, version 22.0 (SPSS, Chicago, Illinois).

### Reported cases in the literature review

A comprehensive literature review revealed 50 reported cases of iliac vein aneurysms. Table 1 illustrates all reported cases of iliac vein aneurysms in the literature.^7-50^, The mean age of reported cases was 41.6 ± 17.8 years (Min = 13; Max: 70). 31 cases (64.6%) were male and 17 cases (35.4%) were female. Mean ages of male and female cases were 44.8 ± 17.8 and 34.5 ± 16.3 years respectively (*P* >0.05).

**Table 1:** Literature review on all previously reported cases of iliac vein aneurysms

**Author(s)**	**Year**	**Age-Sex**	**Location**	**Presentation**	**Etiology**	**Intervention**
Linder^7^	1951	No data	EIV	No data	Secondary to AVF	AR
Cornet et al * (1^st^)^8^	1969	30-M	R-CIV	Limb swelling	Traumatic AVF	No data
Cornet et al * (2^nd^)^8^	1969	50-M	L-EIV	Abdominal mass	Traumatic AVF	AVF closure + AR
Raithel^9^	1972	48-M	L-EIV	Limb swelling	Traumatic AVF	AVF closure
Vaccaro et al^10^	1975	65-M	R-EIV	Limb swelling	Traumatic AVF	Conservative
Parer et al^11^	1984	23-F	L-EIV	Adnexal mass	AVF & Renal transplant	AVF closure
Mansfeld et al^12^	1985	56-M	R-EIV	Limb swelling	Traumatic AVF	AVF closure + AR
Valdes et al^13^	1986	58-M	L-IIV	Abdominal pain	Congenital AVM	Embolization + AR
Tisnado et al^14^	1988	57-M	R-EIV	Venous stasis	Traumatic AVF	AVF closure
Hurwitz & Gelabert^15^	1989	69-M	L-CIV & EIV	Limb pain	Thrombosis	Excision + Bypass
Postma et al^16^	1989	33-M	L-IIV	PE	Primary	Ligation
Gade^17^	1991	13-M	L-EIV	Limb swelling	Congenital IVC hypoplasia	Failed thrombolysis + AR
Salman et al^18^	1994	53-M	L-CIV	Leg ulcers	Traumatic AVF	AVF closure + AR + Bypass
Saito et al^19^	1995	19-M	R-CIV	Abdominal pain	Double IVC	Conservative
Labropoulos et al^20^	1996	34-F	R-CIV & EIV	Limb swelling	Double EIV	Ligation
Alatri & Radicchia ^21^	1997	39-M	B-CIV	Asymptomatic	Primary	Conservative
Petrunić et al^22^	1997	19-M	L-CIV	Limb pain	Primary	AR
Jalaluddin et al^23^	1998	63-F	R-EIV	Hip pain	Primary	Conservative
Fourneau et al^24^	1998	21-F	L-CIV	Asymptomatic	Primary	AR & Bypass
Frikha et al^25^	1999	30-M	R-EIV	Leg ulcers	Traumatic AVF	AVF closure
Al-Damegh^26^	2002	16-M	L-EIV	Shock	Blunt Trauma	Endovascular
Alonso-Perez et al^27^	2002	67-M	B-CIV	Limb swelling	IVC aneurysm	AR
Yoshikawa et al^28^	2002	70-M	L-CIV	Varicose veins	Traumatic AVF	AVF closure
Banno et al^29^	2004	20-F	L-EIV	Asymptomatic	Primary	AR
Cañibano et al^30^	2007	69-M	L-CIV & EIV	Limb swelling	Primary	Conservative
Ysa et al^1^	2008	51-M	R-EIV	Limb pain	Primary	Conservative
Kuhlencordt et al^2^	2008	46-M	L-EIV	Limb swelling	Traumatic AVF	AVF closure + AR + Bypass
Kotsis et al^31^	2009	31-F	L-EIV	Asymptomatic	Primary	AR
Vasquez et al^4^	2009	30-M	L-EIV	Previous trauma	Traumatic AVF	AR & AVF closure
Ysa et al^5^	2010	30-M	R-IIV	Follow-up	Previous DVT	Conservative**
Humphries & Dawson^32^	2010	32-F	B-EIV	Asymptomatic	Primary	Conservative
Takahashi et al^33^	2010	29-F	R-EIV	Right cystic mass	Primary	AR & Patch venoplasty
Tetik et al^34^	2011	34-M	R-EIV	Limb swelling	Traumatic AVF	AVF closure + AR
Zou et al^35^	2011	14-F	L-EIV	PE	Primary	Conservative***
Ghidirim et al^36^	2011	59	R-EIV	Abdominal pain	Primary	AR
Jayaraj & Meissner^37^	2012	37-F	L-EIV	Gluteal pain	Primary	AR
Masood et al^38^	2012	48-M	L-EIV	Previous trauma	Traumatic AVF	AVF closure
Todorov & Hernandez^39^	2013	62-M	L-EIV	Previous trauma	Congenital or AVF	Endovascular
Yoon et al^40^	2013	63-F	L-CIV	Back pain	Primary	Conservative
Hosaka et al^41^	2014	22-F	R-EIV	PE	Primary	AR + Patch venoplasty
Banzic et al^42^	2014	24-F	L-CIV	Thigh skin ulcers	Multiple AVFs	Conservative**
Thompson et al^43^	2015	55-M	L-EIV	Previous trauma	Traumatic AVF	Endovascular
Shah et al^44^	2015	22-F	R-EIV	Arterial emboli	PFO	Endovascular + AR (Hybrid)
Escobar et al^45^	2015	54-F	R-EIV	Incidental	Primary	Conservative
Lucas et al^46^	2015	25-M	L-EIV	Limb swelling	Primary	AR
Park et al^47^	2016	63-F	R-EIV	Rupture	Primary	AR
Audu et al^48^	2017	63-M	L-IIV	Left testis pain	Primary	Endovascular
Lyons et al^49^	2017	24-M	L-CIV	Shock	Blunt Trauma	Conservative
Saddoud et al^50^	2017	61-M	L-EIV	PE	IVC aneurysm	Conservative
DeWane et al^3^	2018	35-F	L-CIV	Asymptomatic	AVF due to spine surgery	Endovascular

AR, aneurysm resection; AVF, arteriovenous fistula; B, Bilateral; CIV, common iliac vein; DVT, deep vein thrombosis; EIV, external iliac vein; IIV, internal iliac vein; L, Left; PE, pulmonary embolism; PFO, patent foramen ovale; R, Right. *Two cases **The patient refused surgery ***The patient underwent pulmonary artery mechanical fragmentation and she also received anticoagulation. Her family refused surgical intervention.

The location of iliac vein aneurysms was studied. The side of the aneurysms was also studied. Tables 2 and 3 demonstrate frequency and percent of iliac vein aneurysms in left and right sides and also in common, external and internal iliac veins. Iliac vein aneurysm was more common in left side in both genders and in total. Also, it was more common in external iliac vein.

**Table 2:** Frequency and percent of iliac vein aneurysms in left and right sides

	**Left side**	**Right side**	**Bilateral**	***P*** value*
Male	20 (64.5%)	9 (29.0%)	2 (6.5%)	>0.05
Female	9 (52.9%)	7 (41.2%)	1 (5.9%)	
Total	29 (60.4%)	16 (33.3%)	3 (6.3%)	

*Chi-square test.

**Table 3:** Frequency and percent of iliac vein aneurysms in common, external and internal iliac veins

	**IIV**	**EIV**	**CIV**	**EIV & CIV**	P value*
Male	4 (12.5%)	17 (54.8%)	8 (25.8%)	2 (6.5%)	> 0.05
Female	0 (0.0%)	12 (70.6%)	4 (23.5%)	1 (5.9%)	
Total	4 (8.3%)	29 (60.4%)	12 (25.0%)	3 (6.3%)	

CIV, common iliac vein; EIV, external iliac vein; IIV, internal iliac vein. *Chi-square test.

Presentation of cases of iliac vein aneurysms were also analyzed. Eight cases (16.7%) were asymptomatic while others presented with limb swelling or pain, signs of venous insufficiency, pulmonary embolism (PE), history of previous trauma, abdominal mass, rupture or shock, back pain, abdominal pain, testicular pain and arterial thrombosis. The most common presentation in men was limb swelling and signs of venous insufficiency. However, most women with iliac vein aneurysm were asymptomatic (*P* < 0.05). Table 4 demonstrates frequency and percent of presentation of cases of iliac vein aneurysms in males, females and in total.

**Table 4:** Frequency and percent of presentations of cases of iliac vein aneurysms

**Presentation**	**Male**	**Female**	**Total**
Asymptomatic	2 (6.5%)	6 (35.3%)	8 (16.7%)
Limb swelling or pain	13 (41.9%)	1 (5.9%)	14 (29.2%)
Abdominal mass	1 (3.2%)	2 (11.8%)	3 (6.3%)
Rupture or shock	2 (6.5%)	1 (5.9%)	3 (6.3%)
Testicular pain	1 (3.2%)	0 (0.0%)	1 (2.1%)
Pulmonary embolism	2 (6.5%)	2 (11.8%)	4 (8.3%)
Arterial thrombosis	0 (0.0%)	1 (5.9%)	1 (2.1%)
Back pain	0 (0.0%)	3 (17.6%)	3 (6.3%)
History of previous trauma	4 (12.9%)	0 (0.0%)	4 (8.3%)
Venous insufficiency signs	4 (12.9%)	1 (5.9%)	5 (10.4%)
Abdominal pain	2 (6.5%)	0 (0.0%)	2 (4.2%)

Etiology of iliac vein aneurysms was also analyzed. 19 cases (39.6%) of reported cases had primary iliac vein aneurysms. Other etiologies consisted of AVF, congenital venous aneurysms, associated venous anomalies and venous thrombosis. The main causes of iliac vein aneurysm in males and females traumatic AVFs and primary aneurysms respectively. This difference was statistically significant (*P* < 0.05). Table 5 demonstrates frequency and percent of etiologies of iliac vein aneurysms in males, females and in total.

**Table 5:** Etiologies of iliac vein aneurysms in reported cases in the literature review

**Etiology**	**Male**	**Female**	**Total**
Primary	7 (22.6%)	12 (70.6%)	19 (39.6%)
Traumatic AVF	16 (51.6%)	0 (0.0%)	16 (33.3%)
Non-traumatic AVF	1 (3.2%)	3 (17.6%)	4 (8.3%)
Congenital	1 (3.2%)	1 (5.9%)	2 (4.2%)
Venous anomaly	4 (12.9%)	1 (5.9%)	5 (10.4%)
Venous thrombosis	2 (6.5%)	0 (0.0%)	2 (4.2%)

AVF, arteriovenous fistula.

Conservative management of iliac vein aneurysm was conducted in 14 patients (28.6%). Resection of aneurysm was reported in 15 patients (30.6%). In addition, resection of venous aneurysm together with AVF closure was reported in six patients (12.2%). AVF closure alone was also reported in six patients (12.2%). Endovascular approach was conducted in five patients (10.2%) and hybrid procedure was conducted in one patient (2.0%). Venous bypass was conducted in three patients following aneurysm resection. In two patients patch venoplasty was considered after aneurysm resection. Simple venorrhaphy was considered in remainder of patients who underwent aneurysm resection.

## Anatomical location of iliac vein aneurysms

The most common site for iliac vein aneurysm is left external iliac vein. This location is the most common site both in males and females. The second common location is common iliac vein (See Tables 2 and 3). The most common etiology of iliac vein aneurysm in males and females is traumatic AVF and primary respectively. Left common iliac vein is located under left iliac artery. Thus, aneurysmal degeneration of iliac vein in external iliac area is more probable either in proximal venous AVF or in primary conditions.

May-Thurner syndrome (MTS) is an anatomical condition resulting in compression of the left common iliac vein between the right common iliac artery and the underlying spine. It may cause subsequent development of a left deep vein thrombosis (DVT).^51^ MTS is more common in women than in men.^52^ The main presentation of MTS is DVT and signs of venous insufficiency in left lower extremity.^53^ However, venous outflow obstruction may result in proximal aneurysmal degeneration in external iliac vein. Our review of iliac vein aneurysms reveals that the most common etiology of iliac vein aneurysms in women is primary type. MTS is more common in women. Thus, primary iliac vein aneurysm in women could be associated with MTS. Therefore, evaluation of aneurysmal degeneration of left external iliac vein is proposed in patients being assessed for MTS.

## Presentation of iliac vein aneurysms

Iliac vein aneurysms could present by lower extremity pain and swelling. Signs of venous insufficiency may occur.^1,6,14^ However, iliac vein aneurysm may be asymptomatic and be found incidentally while the patient being evaluated for abdominal or back pain.^19,40^ In addition, iliac vein aneurysms may mimic adnexal masses.^11,33^

Venous blood stasis in aneurysmal cavity may lead to venous thrombosis.^15^ Following thrombus formation, PE may occur.^16,35,41,50^ Thus, iliac vein aneurysm should be considered in rare differential diagnosis of PE, especially when PE is associated with signs of venous insufficiency in corresponding thrombosed limb. Two cases of aneurysmal related PEs in the literature have been managed by aneurysm exclusion (One case by ligation and one case by resection). Two other cases were managed conservatively.

In addition to PE, iliac vein aneurysm may rupture and lead to profound shock.^26,47,49^ Rupture may occur either in retroperitoneal or intraperitoneal cavity. It is assumed that intraperitoneal free rupture may increase morbidity and mortality. Iliac vein aneurysm rupture should be considered in rare differential diagnosis of retroperitoneal and intraperitoneal bleeding.

## Etiology of iliac vein aneurysms

Iliac vein aneurysms in men are mostly related to a previous AVF. The most common cause of AVFs that lead to aneurysmal venous degenerations are trauma-related fistulas. Arterial flow in iliac venous plexus leads to aneurysmal degeneration in external and common iliac veins.^8-10,14,25,28^ AVFs located distant from the iliac area could cause aneurysmal degeneration of iliac vein. Lack of anterior muscular compression in iliac area leads to aneurysmal degeneration in common and external iliac veins in the cases of distant AVFs in femoral or popliteal regions. Even popliteal AVFs could cause an iliac vein aneurysm.^38^

Most iliac vein aneurysms in women are primary.^23,24^ It could be associated with higher prevalence of MTS in women. However, primary iliac vein aneurysm could be found in men too.^21,22,30^ Primary iliac vein aneurysm should be considered after excluding possible secondary causes especially a history of previous trauma and an AVF. Inferior vena cava (IVC) abnormalities such as IVC hypoplasia and duplication should also be a secondary cause of iliac vein aneurysms.^17,19,27^

## Management of iliac vein aneurysms

Duplex ultrasound, CT venography, MRV and conventional venography could be used for assessment of iliac vein aneurysms.^1,6,48-50^ However, due to the anatomical location of iliac veins inside the pelvic and abdominal cavities, ultrasound may have compromised accuracy. There is not any prospective study in the literature review to propose a single method of choice in the diagnosis of iliac vein aneurysms. Venography seems to reveal the anatomy of iliac vein aneurysms precisely. Saphenous, popliteal and femoral vein accesses may be used in this respect. In the cases of AVF-related iliac vein aneurysms, arteriography may also be used to reveal details of AVF and subsequent venous aneurysm.

Iliac vein aneurysms could lead to fatal complications such as PE, rupture and hemorrhagic shock.^16,26,35,41,47,49,50^ Thus, surgical management of these aneurysms is proposed. Although conservative management of iliac vein aneurysms were reported in the literature review,^21,23,49,50^ this should only be considered in selected cases. Conservative management consists of interval follow-up together with anticoagulation. Surgical management of iliac vein aneurysms is either by open surgical techniques of by endovascular therapies.

Open surgery for iliac vein aneurysm depends of its etiology. In AVF-related aneurysms, simple AVF closure could terminate arterial flow and diminish the aneurysm.^9^ However, aneurysm resection may follow AVF ligation. In primary iliac vein aneurysms, aneurysm resection is considered for open surgical management.^4,8,12^ Following aneurysm resection, venorrhaphy, patch venoplasty^33,41^ or venous bypass^2,18^ are used to restore venous drainage. Traditionally, simple ligation of distal and proximal parts of venous aneurysm may be considered if collateral and superficial venous drainage is sufficient.^20^

Endovascular and hybrid managements of iliac vein aneurysms are reported in six cases in the literature review^3,26,39,43,44,48^ (Table 6). AVF exclusion by arterial stent graft is the proposed method for iliac aneurysms resulting from traumatic AVFs. Then, the size of aneurysm should be followed-up.^3,43^ If open closure of AVF is considered, the venous aneurysm is then could be excluded by a venous graft.^39^ Traumatic iliac vein pseudoaneurysms are managed by trans-catheter embolization.^26^ Primary internal iliac vein aneurysms are managed by coil embolization.^48^

**Table 6:** Endovascular techniques used for iliac vein aneurysm management in the literature review

**Author(s)**	**Year**	**Location**	**Etiology**	**Procedure**
Al-Damegh^26^	2002	L-EIV	Blunt trauma	Trans-catheter embolization
Todorov & Hernandez^39^	2013	L-EIV	Previous AVF*	Venous stent graft
Thompson et al.^43^	2015	L-EIV	Traumatic AVF	Arterial stent graft
Shah et al.^44^	2015	R-EIV	PFO	Embolectomy + Aneurysmectomy
Audu et al.^48^	2017	L-IIV	Primary	Coil embolization
DeWane et al.^3^	2018	L-CIV	AVF**	Arterial stent graft

AVF, arteriovenous fistula; CIV, common iliac vein; EIV, external iliac vein; IIV, internal iliac vein; L, Left; PFO, patent foramen ovale; R, Right. *The AVF was ligated decades ago. **Due to spine surgery.

## Ethical approval

Not applicable.

## Competing interest

The authors declare no conflict of interest.
